# Hospitalizations for infections in primary Sjögren’s syndrome patients: a nationwide incidence study: Severe infections in Sjögren’s syndrome

**DOI:** 10.1080/07853890.2022.2126517

**Published:** 2022-09-29

**Authors:** Radjiv Goulabchand, Alain Makinson, Jacques Morel, Philine Witkowski-Durand-Viel, Nicolas Nagot, Paul Loubet, Camille Roubille, Danièle Noel, David Morquin, Kim Henry, Thibault Mura, Philippe Guilpain

**Affiliations:** a Internal Medicine Department, CHU Nîmes, University Montpellier, Nîmes, France; b University of Montpellier, Montpellier, France; c IRMB, University of Montpellier, INSERM, Montpellier, France; d Department of Infectiology, INSERM U1175, Saint Eloi Hospital and Montpellier University Hospital, Montpellier, France; e Department of Rheumatology, Montpellier University Hospital, Montpellier, France; f PhyMedExp, University of Montpellier, INSERM U1046, CNRS UMR 9214, Montpellier, France; g Department of Internal Medicine and Multi-organic Diseases, St Eloi Hospital, Montpellier University Hospital, Montpellier, France; h Department of Medical Information, Montpellier University Hospital, Montpellier, France; i Department of infectious and Tropical Diseases, CHU Nîmes, University Montpellier, Nîmes, France; j Department of Internal medicine, Lapeyronie Hospital, Montpellier University Hospital, Montpellier, France; k Department of Biostatistics, Clinical Epidemiology, Public Health, and Innovation in Methodology, CHU Nîmes, University Montpellier, Nîmes, France

**Keywords:** Sjögren’s syndrome, infections, pneumonia, intestinal infections, mycobacteria

## Abstract

**Background:**

Primary Sjögren’s syndrome (pSS) is an autoimmune disease with increased risk of infections. Here, we assessed whether pSS patients were at higher risk of hospitalization for community and opportunistic infections.

**Methods:**

We selected newly hospitalized pSS patients between 2011 and 2018, through a nationwide population-based retrospective study using the French Health insurance database. We compared the incidence of hospitalization for several types of infections (according to International Classification for Disease codes, ICD-10) between pSS patients and an age- and sex-matched (1:10) hospitalized control group. We calculated adjusted Hazard Ratios (aHR, 95% CI) adjusted on socio-economic status, past cardiovascular or lung diseases and blood malignancies factors.

**Results:**

We compared 25 661 pSS patients with 252 543 matched patients. The incidence of hospitalizations for a first community infection was increased in pSS patients [aHR of 1.29 (1.22–1.31), *p* < .001]. The incidence of hospitalization for bronchopulmonary infections was increased in pSS patients [aHR of 1.50 (1.34–1.69), *p* < .001, for pneumonia]. Hospitalizations for pyelonephritis and intestinal infections were increased [aHR of 1.55 (1.29–1.87), *p* < .001 and 1.18 (1.08–1.29), *p* < .001, respectively]. Among opportunistic infections, only zoster, and mycobacteria infections (tuberculosis and non-tuberculous) were at increased risk of hospitalization [aHR of 3.32 (1.78–6.18), *p* < .001; 4.35 (1.41–13.5), *p* = .011 and 2.54 (1.27–5.06), *p* = .008, respectively].

**Conclusions:**

pSS patients are at higher risk of hospitalization for infections. The increased risk of hospitalization for mycobacterial infections illustrates the potential bilateral relationship between the two conditions. Vaccination against respiratory pathogens and herpes zoster virus may help prevent some hospitalizations in pSS patients.

**KEY MESSAGES**
- Primary Sjögren’s syndrome (pSS) increases hospitalization risk for community infections: bronchopulmonary, skin, dental, ear–nose–throat, intestinal infections and pyelonephritis.
- Hospitalizations for zoster and mycobacterial infections are also increased in this population.
- Dedicated preventive measures and vaccination campaigns could decrease the burden of infections in pSS patients.

## Introduction

Sjögren’s syndrome (SS) is an autoimmune disease with a wide clinico-pathologic spectrum expanding from an autoimmune exocrinopathy, to systemic disease, and can evolve to B-lymphocyte malignancy [1]. Leading causes of mortality are blood malignancies [1], cardiovascular diseases [2] and infections [3–5], especially bronchopulmonary infections. Infections were responsible for 24% of deaths in one series of 1045 SS patients [1].

The impact of infections on the prognosis and mortality of autoimmune diseases has been widely described in systemic lupus erythematosus (SLE) [6], systemic sclerosis [7] and rheumatoid arthritis [8]. SS differs from these diseases due to the reduced amount of steroids or immunosuppressive treatments used [9] and a pathophysiology potentially involving microbial triggers such as Epstein Barr Virus [10] or mycobacteria [11].

Among all causes of hospitalizations of pSS patients, infections make up around 30% of cases [5], including in intensive care units [12]. However, little is known about the epidemiology of severe infections (i.e. requiring hospitalization) in large cohorts of pSS patients [13]. A better description of the risks of specific infections in pSS patients could help to tailor preventive strategies. We aimed to describe the comparative incidence of hospitalization for community and opportunistic infections between pSS hospitalized patients and matched controls.

## Methods

### Database

We performed a historical paired exposed–unexposed cohort study by analysing data from the French National Hospital discharge database (‘programme de médicalisation des systèmes d‘information’), which covers 99% of hospitalized patients. Available data were: age, sex, entry and discharge dates, discharge diagnoses’ codes according to international classification of diseases (ICD-10), death in hospitalization, and socioeconomic status (specific insurance coverage for low-income people). Information on clinical (systemic involvement of the disease), biological (including autoantibody positivity), pathological data, procedures or treatments (including steroids or immunosuppressive treatments) was not available. We studied a 10-year period between 1 January 2009 and 31 December 2018.

### Study population

We included all patients with a first hospitalization for pSS from 2011. First, we selected all hospitalized patients with at least one ICD-10 code of SS (M350). The date of the first hospitalization was defined as the index date. We excluded all patients with a suspected secondary SS (sSS, i.e. SLE, systemic sclerosis, rheumatoid arthritis, vasculitis, hepatitis C virus etc. Supplementary Table S1), identified through their corresponding ICD-10 codes over the entire study period. Patients with a first code of SS in 2009 or 2010 were excluded, to have at least 2 years of available medical history of hospitalization prior to the index date. This cohort was described in previous studies [14]. We randomly constituted a control group (1:10) of hospitalized patients matched on age, sex and index date within the same database (excluding pSS subjects). We excluded patients who died in hospitalization within the 90 days after the index date in both groups, to avoid selection of patients with acute severe and critical illnesses at the index date. For each studied infection, all subjects with prior hospital admission for the condition before the incidence study period, and their matched patients, were excluded.

**Table 1.** Characteristics of hospitalized primary Sjögren’s syndrome patients and their matched controls, and adjustment covariates.

	Sjögren’s syndrome patients (*n* = 25 661)		Matched controls (*n* = 252 543)		
	Number of patients	% of pSS patients	Number of matched patients	% of matched patients	Comparison (*p* value)
Sex (female, *n*, %)	22 489	87.66	224 887	87.65	.995
Low socio-economic status (*n*, %)	1224	4.77	8482	3.31	<.001
Mean age at index date (mean, *SD*)	60.2	± 16.3	60.0	± 16.3	.075*
Mean number of hospitalizations before index date (mean, *SD*)	3.7	± 9.0	0.21	± 1.1	<.001*
Annual rate of hospitalisations before index date (*n*, %)					
≤ 0.25 per year	10 245	39.93	244 107	95.15	<.001
between 0.25 and 0.5 per year	5959	23.23	8679	3.38	–
between 0.5 and 1 per year	5430	21.16	2807	1.09	–
between 1 and 5 per year	3704	14.44	908	0.35	–
>5 per year	318	1.24	59	0.02	–
Deaths (incidence,^a^ CI)	14.4	[13.7–15.2]	10.5	[10.3–10.7]	<.001
Follow-up time after index date [median, (IQR), years]	3.96	[1.96–5.96]	3.96	[1.96–6.04]	.003*
Conditions reported during hospitalizations stays before the 90th day after index date (used as adjustment covariates)					
Hypertension	274	1.07	876	0.35	<.001
Diabetes	656	2.56	2818	1.12	<.001
Obesity	294	1.15	1393	0.55	<.001
Cardiovascular diseases	1361	5.30	8281	3.28	<.001
Dialysis	83	0.32	214	0.08	<.001
Interstitial lung disease	695	2.71	154	0.06	<.001
Chronic obstructive pulmonary disease	442	1.72	1202	0.48	<.001
Blood malignancies	566	2.21	1263	0.50	<.001
Neuropsychiatric disorders (anxiety, depression, dementia)	667	2.60	2529	0.99	<.001

CI: confidence interval; pSS: primary Sjögren’s syndrome; *n*: number; SD: standard deviation.

^a^Incident deaths per 1000 person–years.

All comparisons were performed with *χ*^2^ test except those with an asterisk (*), performed with Wilcoxon–Mann–Whitney rank-sum test.

### Outcomes

We recorded the first occurrence of hospitalization for all pooled community infections, and all pooled opportunistic infections, in pSS and controls. We then recorded the first hospitalization for each infection within these groups. Conditions were identified according to their ICD-10 codes (Supplementary Table S2). Community infections of interest were: bronchopulmonary infections (pneumonia, bronchitis, flu); urinary tract infections, pyelonephritis, prostatitis; meningitis; skin infections (erysipelas, dermo-hypodermitis and skin abscesses); endocarditis and other sepsis (staphylococcus, *Hemophilus influenzae*, anaerobes, anaerobes or unspecified sepsis); phlegmon; dental and Ear–Nose–Throat (ENT) infections; abdominal infections (anorectal abscess, intestinal abscess or fistula, peritonitis, bacterial intestinal infections, cholecystitis, diverticulitis); pelvic infections (salpingitis and oophoritis, acute parametritis and pelvic cellulitis, diseases of Bartholin’s gland, vulvo-vaginitis); arthritis and bone infections (osteomyelitis, infection and inflammatory reaction due internal orthopaedic prosthetic devices, implants and grafts, infective spondylopathies and discitis).

**Table 2.** Incidence of first hospitalization for community infections in hospitalized primary Sjögren’s syndrome patients and controls.

	pSS patients				Matched controls									
	Incident cases^a^	Py	Incidence^a^	CI	Incident cases^a^	Py	Incidence^a^	CI	Crude HR	Crude CI	Crude *p* value	aHR	Adjusted CI	Adjusted *p* value
At least one incident hospitalization for one community infection	2254	82 804	27.22	[26.1–28.34]	12,829	782,414	16.40	[16.12–16.68]	1.55	[1.68–1.73]	.000	**1.29**	**[1.22–1.38]**	**<.001**
Pneumonia	699	98,060	7.13	[6.60–7.70]	3335	974,095	3.42	[3.30–3.54]	2.08	[1.91–2.26]	.000	**1.50**	**[1.34–1.69]**	**<.001**
Bronchitis	229	101,615	2.25	[1.96–2.54]	866	1,012,607	0.86	[0.80–0.92]	2.67	[2.30–3.10]	.000	**1.70**	**[1.36–2.11]**	**<.001**
Flu	70	102,554	0.68	[0.52–0.84]	267	1,021,275	0.26	[0.23–0.29]	2.64	[2.02–3.44]	.000	**1.98**	**[1.32–2.97]**	**.001**
Urinary tract infection	131	102,323	1.28	[1.06–1.50]	807	1,017,813	0.79	[0.74–0.84]	1.63	[1.35–1.96]	.000	0.98	[0.74–1.30]	.881
Pyelonephritis	305	101,015	3.02	[2.68–3.36]	1502	1,004,922	1.49	[1.41–1.57]	2.01	[1.77–2.27]	.000	**1.55**	**[1.29–1.87]**	**<.001**
Prostatitis^a^	37	11,750	3.15	[2.14–4.16]	262	117,224	2.24	[1.97–2.51]	1.43	[1.01–2.03]	.044	1.03	[0.68–1.57]	.883
Meningitidis	6	102,839	0.06	[0.01–0.11]	28	1,023,721	0.03	[0.02–0.04]	2.02	[0.84–4.89]	.118	1.57	[0.32–7.77]	.582
Skin abscess	82	102,306	0.80	[0.63–0.97]	609	1,016,140	0.60	[0.55–0.65]	1.33	[1.06–1.68]	.014	0.97	[0.72–1.30]	.966
Skin infections (erysipela, dermo-hypodermitis)	18	102,745	0.18	[0.10–0.26]	72	1,022,931	0.07	[0.05–0.09]	2.53	[1.5–4.26]	.000	**3.54**	**[1.54–8.18]**	**.003**
Endocarditis	28	102,793	0.27	[0.17–0.37]	120	1,023,147	0.12	[0.10–0.14]	2.34	[1.54–3.54]	.000	1.71	[0.95–3.06]	.071
Other sepsis	124	102,350	1.21	[1.00–1.42]	671	1,018,885	0.66	[0.61–0.71]	1.82	[1.50–2.21]	.000	1.04	[0.78–1.37]	.813
Phlegmon	44	102,583	0.43	[0.3–0.56]	290	1,019,703	0.28	[0.25–0.31]	1.47	[1.07–2.04]	.019	1.29	[0.85–1.96]	.236
Dental and ENT infections	198	100,957	1.96	[1.69–2.23]	1053	1,000,519	1.05	[0.99–1.11]	1.85	[1.59–2.15]	.000	**1.27**	**[1.04–1.56]**	**.021**
Intestinal infections	992	93,771	10.58	[9.92–11.24]	6672	903,196	7.39	[7.21–7.57]	1.42	[1.32–1.54]	.000	**1.18**	**[1.08–1.29]**	**<.001**
Pelvic infections	51	102,369	0.50	[0.36–0.64]	407	1,016,130	0.40	[0.36–0.44]	1.25	[0.93–1.67]	.138	1.08	[0.74–1.57]	.699
Arthritis/bone infections	99	102,260	0.97	[0.78–1.16]	625	1,017,475	0.61	[0.56–0.66]	1.54	[1.24–1.91]	.000	1.02	[0.76–1.36]	.915

ENT, ear–nose–throat; pSS, primary Sjögren’s syndrome; CI, confidence interval; py, person–years; #, number of incident cases per 1000 person–years; HR, hazard ratio; aHR, adjusted HR.

^a^Calculation performed only among male patients.

Other sepsis: staphylococcus, *Hemophilus influenzae*, anaerobes, anaerobes or unspecified sepsis.

Intestinal infections: anorectal abscess, intestinal abscess or fistula, peritonitis, bacterial intestinal infections, cholecystitis, diverticulitis.

Arthritis and bone infections: arthritis, osteomyelitis, infection and inflammatory reaction due internal orthopaedic prosthetic devices, implants and grafts, infective spondylopathies and discitis.

Pelvic infections: Salpingitis and oophoritis, Acute parametritis and pelvic cellulitis, Diseases of Bartholin’s gland, vulvo-vaginitis.

Adjustment factors included annual hospitalization rate and previous report (before incidence study period) of low socio-economic status, diabetes, hypertension, obesity, cardiovascular diseases, dialysis, blood malignancies and neuro-psychiatric disorders (dementia, depression, anxiety). For bronchopulmonary infections (pneumonia, bronchitis, influenza), adjustment factors also included baseline mention of chronic obstructive pulmonary disease (COPD) and interstitial lung disease (ILD).

Bold face values are applied to lines associated with infections statistically more incident (*p*<0.05) in pSS patients.

For opportunistic infections, we recorded: herpes, zoster, chicken pox, cytomegalovirus, non-tuberculous mycobacteria (NTM), tuberculosis, listeria, salmonella *non-typhii*, pneumocystis, toxoplasmosis, histoplasmosis, candidiasis and aspergillosis.

We also studied the comparative mortality incidence in hospital for the following infections: pneumonia, pyelonephritis, intestinal infections, and dental and ENT infections.

### Covariates

We searched for existing conditions potentially influencing infection risks prior to the 90th day after the index date: low socioeconomic status (LSS), annual rate of hospitalization before the index date, hypertension, diabetes, obesity, cardiovascular diseases, dialysis, neuropsychiatric disorders (dementia, depression or anxiety), chronic obstructive pulmonary disease (COPD), interstitial pneumonitis and lung fibrosis (interstitial lung disease, ILD), and blood malignancies (lymphoma, Waldenström macroglobulinaemia, multiple myeloma, and leukemia, Supplementary Table S1).

### Statistical analysis

Conditions were compared between cases and controls using χ^2^, Fisher exact test, or Mann–Whitney rank-sum test when appropriate. The incidences of overall community and opportunistic infections, and the incidences of each infection, were calculated in each exposure group, with their 95% confidence intervals (CI), after excluding patients who were already hospitalized for the studied condition before the 90th day after the index date. A survival analysis by Cox proportional hazard models, with stratification on the matched subjects, was then performed to compare incidence between exposure groups. Follow-up time was calculated as the interval between the index date and the date of the first event detected, or until December 2018, whichever occurred first. Follow-up of patients who died in hospital before December 2018, without any event, were censored at the date of death to take into account competitive risks. Analysis was made on the available data (no imputation of missing data).

For the study of the incidence of infections, we first built a crude statistical model. Then, to take into account potential confounding factors [3] influencing the incidence of infections, we built an adjusted model including LSS, annual hospitalization rate, history of hypertension, diabetes, obesity, dialysis, cardiovascular diseases, blood malignancies and neuropsychiatric disorders. For the study of hospitalizations for bronchopulmonary infections (pneumonia, bronchitis, flu, tuberculosis, non-tuberculous infections, pneumocystis and aspergillosis), we also included in our adjustment factors history of COPD and ILD. To study whether the association between bronchopulmonary infections and pSS was mediated by the onset of ILD, we performed an additional model including this parameter as a time-dependent covariate.

Statistical analyses were performed at the conventional two-tailed α level of 0.05 using Statistical Analysis Systems Enterprise Guide 7.1 (SAS Institute, Cary, NC).

### Ethics

French National Hospital discharge database is an anonymized national health insurance database and studies on anonymized national health insurance database are authorized by the National Commission of Information Technology and Liberty (CNIL). No specific consent or ethical approval was thus needed for this study (https://www.cnil.fr/fr/declaration/mr-005-etudes-necessitant-lacces-aux-donnees-du-pmsi-etou-des-rpu-par-les-etablissements).

## Results

### Study population

The characteristics of the study population are shown in Table 1. We identified 25,661 hospitalized patients with pSS and 252,543 age- and sex- matched hospitalized controls from the French National Health insurance database (Supplementary Figure S1). The population consisted of 87.7% of female patients, with a mean age of 60.0 (±16.3) years and a median follow-up time of 3.96 years. We observed a higher proportion of blood hypertension, diabetes, obesity, cardiovascular diseases, dialysis, blood malignancies and neuropsychiatric conditions in pSS than controls. Moreover, there was a higher proportion of lung diseases (ILD: 2.71% versus 0.06%, and COPD: 1.72% versus 0.48%) among pSS patients.

**Figure 1.**
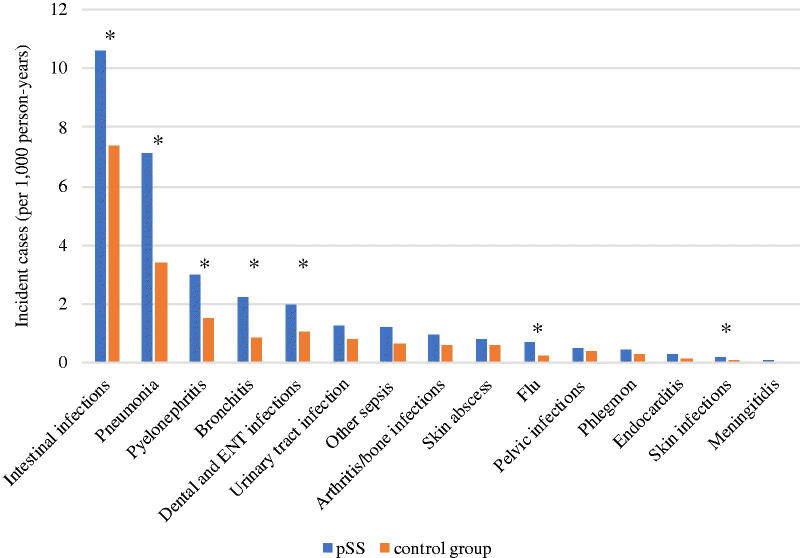
Comparative incidence of hospitalization for community infections in primary Sjögren’s syndrome patients versus matched control. pSS: primary Sjögren’s syndrome patients; ENT: ear-nose-throat. The asterisk * marks the infections with statistically different incidences between pSS patients and matched controls.

Infections reported during hospitalization before the incidence study period are listed in Supplementary Table S3.

**Table 3.** Incidence of first hospitalization for opportunistic infections in hospitalized primary Sjögren’s syndrome patients and controls.

	pSS patients				Matched controls									
	Incident cases^#^	Py	Incidence^#^	CI	Incident cases^#^	Py	Incidence^#^	CI	Crude HR	Crude CI	Crude *p* value	aHR	Adjusted CI	Adjusted *p* value
At least one incident hospitalization for one opportunistic infection	104	101,984	1.02	[0.82–1.22]	383	1,016,040	0.38	[0.34–0.42]	2.76	[2.22–3.43]	.000	**1.98**	**[1.46–2.69]**	**<.001**
Herpes	3	102,843	0.03	[0–0.06]	21	1,023,667	0.02	[0.01–0.03]	1.45	[0.43–4.88]	.549	0.46	[0.03–6.49]	.567
Zoster	24	102,738	0.23	[0.14–0.32]	78	1,023,062	0.08	[0.06–0.1]	3.08	[1.94–4.89]	.000	**3.32**	**[1.78–6.18]**	**<.001**
Chicken pox	1	102,895	0.01	[0.00–0.03]	6	1,024,287	0.01	[0–0.02]	1.67	[0.20–13.8]	.636	2.60	[0.11–64]	.559
Cytomegalovirus	4	102,874	0.04	[0–0.08]	19	1,024,114	0.02	[0.01–0.03]	2.09	[0.71–6.13]	.182	0.35	[0.06–2.22]	.265
Non-tuberculous mycobacteria	11	102,843	0.11	[0.05–0.17]	17	1,023,996	0.02	[0.01–0.03]	6.13	[2.87–13.1]	.000	**4.35**	**[1.41–13.5]**	**.011**
Tuberculosis	14	102,622	0.14	[0.07–0.21]	74	1,021,360	0.07	[0.05–0.09]	1.95	[1.09–3.46]	.024	**2.54**	**[1.27–5.06]**	**.008**
Listeria	2	102,895	0.02	[0.01–0.05]	4	1,024,300	0.00	[0–0]	4.48	[0.82–24.47]	.084	6.47	[0.77–54.5]	.086
Salmonella (*non-typhii*)	8	102,834	0.08	[0.03–0.13]	30	1,023,748	0.03	[0.02–0.04]	2.71	[1.23–5.94]	.013	1.51	[0.44–5.18]	.517
Pneumocystis	7	102,859	0.07	[0.02–0.12]	29	1,024,119	0.03	[0.02–0.04]	2.42	[1.06–5.54]	.037	1.33	[0.37–4.83]	.661
Candidiasis	12	102,809	0.12	[0.05–0.19]	62	1,023,597	0.06	[0.04–0.08]	2.04	[1.09–3.81]	.025	0.96	[0.34–2.71]	.943
Aspergillosis	20	102,780	0.19	[0.11–0.27]	50	1,023,482	0.05	[0.04–0.06]	3.94	[2.34–6.64]	.000	1.68	[0.62–4.57]	.307

pSS: primary Sjögren’s syndrome; CI: confidence interval; py: person–years; #: number of incident cases per 1000 person–years; HR: hazard ratio; aHR: adjusted HR.

Adjustment factors included annual hospitalization rate and previous report (before incidence study period) of low socio-economic status, diabetes, hypertension, obesity, cardiovascular diseases, dialysis, blood malignancies and neuro-psychiatric disorders (dementia, depression, anxiety). For bronchopulmonary infections (tuberculosis, non-tuberculous mycobacteria, pneumocystis and aspergillosis), adjustment factors also included baseline mention of chronic obstructive pulmonary disease (COPD) and interstitial lung disease (ILD).

Bold face values are applied to lines associated with infections statistically more incident (*p*<0.05) in pSS patients.

### Incidence of community infections

The 2254 pSS patients (8.78%) experienced at least one hospitalization for a community infection within the study period (Table 2). After adjustment, the incidence of hospitalizations for a first community infection was increased in pSS patients compared with matched controls [aHR = 1.29, 95% CI (1.22–1.31), *p* < .001]. The four leading causes of incident hospitalizations for infections in pSS patients were: (i) intestinal infections; (ii) bronchopulmonary infections; (iii) pyelonephritis and (iv) dental and ENT infections. Figure 1 shows the comparative incidence of hospitalization for specific subgroups of community infections.

The incidence of hospitalization was increased for intestinal infections in pSS patients aHR = 1.18 [95% CI (1.08–1.29), *p* < .001]. Similarly, pSS patients had a significantly higher incidence rate of hospitalization for bronchopulmonary infections compared with matched controls: aHR were 1.50 [95% CI (1.34–1.69), *p* < .001] for pneumonia, 1.70 [95% CI (1.36–2.11), *p* < .001] for bronchitis and 1.98 [95% CI (1.32–2.97), *p* < .001] for flu. When integrating ILD as a time-dependent covariate in our bronchopulmonary infections survival model, the association between pSS and pneumonia, bronchitis and flu remained similar aHR = 1.48 [95% CI (1.32–1.68), *p* < .001]; aHR = 1.70 [95% CI (1.36–2.11), *p* < .001]; aHR = 1.97 [95% CI (1.32–2.96), *p* < .001], respectively). Incident hospitalization rates for pyelonephritis, skin infections and dental and ENT infections were also increased in pSS patients: aHR 1.55 [95% CI (1.29–1.87), *p* < .001]; 3.54 [95% CI (1.54–8.18), *p* = .003] and 1.27 [95% CI (1.04–1.56), *p* = .021], respectively.

### Incidence of opportunistic infections

The 104 pSS patients (0.04%) experienced at least one opportunistic infection over the study period. The incidence of hospitalizations for a first opportunistic infection was increased in pSS patients compared with matched controls [aHR = 1.98, 95% CI (1.46–2.69), *p* < .001]. Hospitalizations for zoster were increased in the pSS patients [aHR = 3.32, 95% CI: (1.78–6.18), *p* < .001]. Incident hospitalizations for mycobacteria were increased in pSS patients [NTM: aHR = 4.35, 95% CI: (1.41–13.5), *p* = .011; and tuberculosis: aHR = 2.54, 95% CI: (1.27–5.06), *p* = .008, Table 3]. After integrating ILD as a time-dependent covariate in our model, the comparative incidence of NTM and tuberculosis did not change [aHR = 4.37, 95% CI: (1.41–13.5), *p* = .011]; aHR = 2.55 [95% CI: (1.28–5.09), *p* = .008]. Aspergillosis incident rate of hospitalization was higher in pSS patients [aHR = 3.94, 95% CI: (2.34–6.64), *p* < .001], but this association disappeared when integrating ILD as an adjustment factor [aHR = 1.68, 95% CI: (0.62–4.57), *p* = .307]. Notably, the incident hospitalization rate for ILD was increased in pSS [aHR = 9.04, 95%CI: (6.94–11.80), *p* < .001]. There was no incident toxoplasmosis in both groups, and 1 histoplasmosis in control group.

### Mortality

The incidence of in-hospital mortality of pSS patients was not different from that of controls for each group of main infection (pneumonia, pyelonephritis, intestinal infections or dental and ENT infections), regardless of the adjustment factors included.

## Discussion

Our nationwide database study highlights the burden of infections on the hospitalizations of pSS patients in comparison with matched hospitalized patients. We showed an increased risk for hospitalization for community infections, especially bronchopulmonary, intestinal, pyelonephritis, and dental and ENT infections, and for some opportunistic infections (zoster and mycobacteria). Our results are in line with the literature. Among 69,239 hospitalizations of pSS US patients, leading causes of infections were pneumonia, sepsis, soft tissue, skin and subcutaneous infections, and urinary tract infections, while opportunistic infections only involved 3% of subjects [3]. However, to our knowledge, the increased incidence of intestinal infections has never been reported and needs further confirmation.

### Factors associated with community infections

Sicca syndrome may be partially responsible for the higher incidence of dental and ENT infection, skin infections, pyelonephritis (as a consequence of vaginal dryness), and bronchopulmonary infections. The higher rates of intestinal infections may arise from functional digestive disorders and gastroparesia [15], and gut microbial overgrowth (exacerbated by sicca syndrome) [16].

### Opportunistic infections

Although absolute numbers of recorded infections were relatively low, we found increased hospitalization incidence in pSS patients for zoster and mycobacteria. A Taiwanese study including 18 000 pSS patients showed that pSS patients had the lowest incidence of opportunistic infections compared with other auto-immune diseases (dermato-myositis, scleroderma or rheumatoid arthritis [9]). Even for herpes and zoster infection, our incidence rate was lower than previously published in pSS and auto-immune disease populations [9, 17]. This is likely due to only considering inpatients, and to the lower burden of immunosuppressants (especially glucocorticoids) in pSS [9].

We found an increased risk of hospitalization for tuberculous and NTM infections in pSS patients. Chang et al. [18] also found an increased risk of tuberculosis infection in 4822 Taiwanese pSS patients, and Chao et al. [19] observed an increased risk of NTM in 6554 incident Taiwanese SS patients after adjusting for a comorbidity index, glucocorticoids and immunosuppressants use. In a nationwide study involving 5751 pSS patients, an association between a history of NTM and incident pSS was found [20]. The role of sex hormone deprivation on mycobacteria and pSS onset has been proposed [21, 22]. An alternative immunological hypothesis based on a long-lasting immune reaction against mycobacteria has been suggested in pSS patients, through a higher prevalence of anti-hsp65 antibodies (targeting mycobacterium antigen) [23]. The role of TNFAIP3 (TNF-alpha-induced Protein 3, modulating NF-kB activation) has also been suggested in both pSS-related lymphomas [24] and mycobacterial susceptibility [25].

Interestingly, we found a higher incidence of hospitalization for ILD in pSS patients, as previously reported [26, 27], which may have modulated the infectious respiratory risks in pSS patients. In our study, the over-risks of pneumonia, bronchitis, flu, NTM and tuberculosis in pSS patients were not modified when including ILD as a time-dependent covariate. Conversely, the relation between SS and aspergillosis may be partly mediated by the increased risk of ILD in pSS patients. Several pathophysiological mechanisms could be involved: (i) ILD may lead to bronchopulmonary damage, facilitating aspergillosis colonization and survival; (ii) local mucosal-immune surveillance may be impaired and (iii) immunosuppressant treatments of ILD, including glucocorticoids, may further weaken immune defences [27].

We did not find any over-risk of hospitalization for pneumocystis in pSS patients. This contrasts with the excess incidence of pneumocystis of 6.68 (4.03–11.07) in 23 048 pSS patients, in the study by Hsu et al. [28]. These discrepancies may partially be explained by the potential confounding factors involved in the models, such as glucocorticoids and immunosuppressants use, or ILD [28].

### Prevention of higher infection risks

Maintaining good oral health is essential in pSS patients to prevent dental and ENT infections. Ensuring proper skin moisturizing and a good vaginal trophicity may also prevent urinary tract infections and pyelonephritis, especially among postmenopausal patients.

Bronchopulmonary infections and ENT infections could also be reduced through appropriate vaccination, such as annual flu and anti-pneumococcal vaccines. In a previous study, Morel et al. [29] described that only 31.5% of their series of 111 pSS patients were vaccinated against flu, and 11.7% against pneumococcus. Vaccination is probably efficient within pSS population, as shown by the post-vaccination immune response of anti-influenza IgG levels in pSS patients [30], and the anti-pneumococcal response in 15 patients with pSS [31].

### Strengths and limitations

Our methodological approach presents some limits. We only collected events in hospitalized patients as we wanted to focus on the most severe cases. Deaths occurring outside hospitalization were not recorded. Since the diagnoses were based on ICD-10 codes, some patients could have been misclassified for pSS diagnosis or for infections. We could not integrate all the potential cofounding factors for infection risk such as tobacco and alcohol use, and glucocorticoids or immunosuppressants exposure. Indeed, the burden of immunosuppressants in pSS could affect infectious risk, especially in some subgroups of patients with active disease (such as ILD) [9, 28]. However, considering a recent study involving 134 French pSS patients, this impact may be limited since only 17% were treated by steroids and 14% by immunosuppressive drug [32]. Thus, we included the available comorbid conditions (e.g. obesity, cardio-vascular diseases, lung conditions) in our statistical models, since these comorbidities could alter the onset of infections in both groups. Finally, we selected controls who were hospitalized for another reason than pSS. It is therefore probable that controls were less ‘healthy’ than the general population, weakening the strength of identified associations and reinforcing our conclusions about the increased risk of some infections in pSS patients.

## Conclusions

In this large nationwide study, hospitalized pSS patients showed a higher risk of hospitalization for community infections, especially bronchopulmonary infections and intestinal infections, compared with matched controls. Likewise, incidence of opportunistic infections was increased, and a potential bilateral relationship between SS and mycobacteria is suggested. A targeted vaccination campaign against respiratory pathogens and strict management of sicca syndrome could be beneficial in reducing this risk.

## Data Availability

The datasets used and analysed during the current study are available from the corresponding author on reasonable request.
